# Post-conflict acute stress reactions in Kazakhstan in the aftermath of January 2022 unrests: A national survey

**DOI:** 10.1016/j.heliyon.2023.e21065

**Published:** 2023-10-21

**Authors:** Natalya Glushkova, Alexandr Ivankov, Varvara Trenina, Ainash Oshibayeva, Zhanna Kalmatayeva, Zhanar Temirbekova, Saltanat Mamyrbekova, Kairat Davletov, Zaituna Khismetova, Almagul Kauysheva, Ardak Auyezova, Marat Zhanaspayev, Lyudmila Pivina, Gulbakit Koshmaganbetova, Ardak Nurbakyt, Gulzat Sarsenbayeva, Zhanat Sadibekova, Meruert Gazaliyeva, Mukhtar Kulimbet, Diana Kalmatayeva, Aliya Zhylkybekova, Zhansaya Nurgaliyeva, Kassymkhan Sultanbekov, Daniil Semenov, Marina Izmailovich, Oxana Tsigengagel, Kerim Munir

**Affiliations:** a Department of Epidemiology, Biostatistics and Evidence-Based Medicine, Al-Farabi Kazakh National University, Almaty, Kazakhstan; b Independent Researcher, Almaty, Kazakhstan; c Department of Public Health, Semey Medical University, Semey, Kazakhstan; d Rector Office, Khoja Akhmet Yassawi International Kazakh-Turkish University, Turkestan, Kazakhstan; e Academic Office, Asfendiyarov Kazakh National Medical University, Almaty, Kazakhstan; f Scientific Office, Asfendiyarov Kazakh National Medical University, Almaty, Kazakhstan; g Rector Office, Kazakhstan Medical University Higher School of Public Health, Almaty, Kazakhstan; h Department of Traumatology, Semey Medical University, Semey, Kazakhstan; i Department of Internal Medicine, Semey Medical University, Semey, Kazakhstan; j Department of Master's and Doctoral Studies, West Kazakhstan Marat Ospanov Medical University, Aktobe, Kazakhstan; k Department of Public Health, Asfendiyarov Kazakh National Medical University, Almaty, Kazakhstan; l Department of Social Health Insurance and Public Health, South Kazakhstan Medical Academy, Shymkent, Kazakhstan; m Dean Office of the School of Medicine, Karaganda Medical University, Karaganda, Kazakhstan; n Department of General Psychology, Faculty of Philosophy and Political Science, Al-Farabi Kazakh National University, Almaty, Kazakhstan; o Astana IT University, Astana, Kazakhstan; p Department of Internal Diseases, Karaganda Medical University, Karaganda, Kazakhstan; q Developmental Medicine Centre, Boston Children's Hospital and Departments of Psychiatry and Paediatrics, Harvard Medical School, Boston, USA

**Keywords:** Acute stress reactions, Anxiety symptoms, Civil disturbances, Riots, Violence, Kazakhstan

## Abstract

**Background:**

In January 2022 Kazakhstan experienced unprecedented nationwide protests that quickly turned to violent riots. Although the number of individuals affected physically by the disturbances were cited, the emotional toll of the events remains undetermined. The aim of this study was to evaluate the comparative rates of acute stress reactions in Kazakhstan in the aftermath of the unrests.

**Methods:**

A cross-sectional, population-based online survey was conducted one month after the start of the disturbances. The study questionnaire were completed anonymously and included the Primary Care PTSD Screen for DSM-5 (PC-PTSD-5) and the General Anxiety Disorder-7 (GAD-7), as well as socio-demographic and event exposure information.

**Results:**

Of the 7021 people who initially agreed to participate, 6510 were able to complete the full survey. For a cut-off of ≥3 on the PC-PTSD-5, 14.8 % of the study participants exhibited symptoms. With a cut-off of ≥4, this percentage reduced to 4.6 %. Participants from Almaty city and Almaty region who experienced the most extensive disturbances showed a doubled prevalence compared to the national level (30.0 % for a cut-off of ≥3, and 10.1 % for a cut-off of ≥4). At the national level, the prevalence of anxiety symptoms, defined as a score of ≥10 on the GAD-7, stood at 10.9 %. This prevalence decreased to 4.2 % when considering a cut-off of ≥15.

**Conclusion:**

Health authorities of riot-affected areas ought to be aware of the population level mental health impact of the civil conflict and consider provision of targeted interventions to mitigate the long-term consequence of these lifespan disorders, while also seeking for the peaceful resolution of the ensuing conflicts.

## Highlights

• Population of Almaty had the highest rate of emotional distress.
• People under the age of 25 years had lower rate of emotional distress.
• Kazakhstan needs to provide mental health interventions.

## 1 Introduction

On January 2, 2022, a series of mass protests began in western provinces in Kazakhstan following a sharp increase in gas prices precipitated by the government's decision to lift the price cap. The protests quickly spread nationally across the country, with demonstrations turning into violent riots, especially affecting the populations in cities, and especially, in Almaty, the nation's largest city and its cultural and commerce center, with a population of 2 million. A state of emergency was declared by the President Tokayev on January 5, 2022 and the country's government resigned [1]. In Almaty, the buildings of the National Security Committee, Presidential palace and city mayor were stormed and set aflame. Government buildings were also attacked in several other cities. President Tokayev requested the Collective Security Treaty Organization for assistance with special forces from Russia, Armenia, Belarus, Kyrgyzstan and Tajikistan arriving in Kazakhstan on 6 January 6, 2022. The order was restored in all regions of the country on January 7, 2022 and only intermittent protests were seen thereafter, with the state of emergency lasted through 19 January 2022 [2].

Individuals who experience conflict-related traumatic events are more likely to have adverse mental health outcomes compared to those without experience of such events. Furthermore, many experience such mental disorders for the first time in the aftermath of conflict-related trauma exposures [3]. The detrimental effects on mental health of such conflicts are surpassed probably only by war [4]. Individuals with pre-existing vulnerability, those who are separated, divorced, or widowed, elderly, and those with comorbid chronic physical health conditions are at greater risk for developing post-conflict mental health sequalae. These emotions are most prevalent among individuals who directly experience traumatic events, but also occur among those who monitor unrest via television or internet, as well as interaction with others, as a result of their “community spillover effect,” enhanced by sound and sight including varying levels of witnessing of the proximal stories of among family, friends, neighbors involved in direct clashes.

A range of post-conflict related mental health problems have been described and include acute and post-traumatic stress disorders (ASD and PTSD), anxiety, depression, mood and sleep disturbances, substance use and impulse-control disorders including suicide [5]. In general, stress reactions can develop in response to any traumatic event and include a spectrum of psychological symptoms that comprise intrusive and uncontrollable anxious and fearful thoughts about the event, avoidance behaviors, as well as flashbacks, and nightmares. ASD commonly develops shortly after a traumatic event and typically lasts up to one month, while PTSD can sometimes be a progression from ASD, developing after one month following the traumatic events. In post-conflict circumstances the ASD and PTSD symptoms are closely interrelated with generalized anxiety disorder (GAD) [6].

A limited number of individuals with post-conflict related mental disorder seek help, depending on the type and severity of their condition [7]. Average delay in treatment from first onset of the symptoms to seeking treatment vary from months to many years and are most ostensibly delayed for anxiety and substance use related disorders [8]. Many seek help for physical conditions e.g., back or neck pain, headaches, and arthritis, and many mental health related distress continue unrecognized. The situation is particularly salient in low- and middle-income global regions with limited availability of mental health services. Thus, there is a need both for timely recognition and remediation of post-conflict related mental health problems.

Almaty city and provinces of south Kazakhstan were the epicenter of the most violent unrest. The city was overwhelmed by chaos as the airport, police offices, TV stations, and many businesses were stormed or set on fire and looting was widespread. Besides, there was no internet access and radio stations and TV channels broadcasting was also blocked, which increased the psychological tension. Two hundred and twenty-five people were killed during the events and 19 of them were police officers. The number of people arrested approached 10,000 and around 4500 individuals were injured [9]. Although the number of physically impaired is approximately known, there is no evidence considering the number of people who experienced mental distress. The aim of this study was to evaluate the rates of PTSD and GAD in Kazakhstan following January 2022 unrest by means of nationwide population-based survey. A secondary goal was to assist policymakers in planning of mental health services for the victims of violent unrest.

## 2 Materials and methods

### 2.1 Study proceedings

A cross-sectional, population-based online survey was anonymously conducted, between 24–31 January shortly after civil disturbances. A structured questionnaire for the Google survey tool was developed and the link shared with the public via social media, e.g., via Facebook, Instagram, Telegram, What's App, gaining momentum quickly as interest and visibility grew. The questionnaire was kept short, so that the survey could be completed in approximately 10 min. The rationale for online survey approach was the need to reach a maximal population over a short period of time proximal to the January events. The inclusion criteria for survey participation in the online survey were: (i) age 18 years and older; (ii) residence in Kazakhstan at the time of January 2022 unrest; (iii) internet access; and (iv) informed consent. Of the 7021 people who indicated their agreement to participate in the online survey, 6510 were able to complete the entire survey (92.7 %).

### 2.2 Ethics statement

The study protocol was considered by the Ethics Committee of the Kazakhstan Medical University, Higher School of Public Health, Almaty, which granted the permission (Protocol 157 dated January 22, 2022).

### 2.3 Evaluation of acute stress reactions

The Diagnostic and Statistical Manual of Mental Disorders, Fifth Edition (DSM-5), establishes specific criteria for diagnosing both ASD and PTSD. In the case of ASD, symptoms emerge shortly after exposure to a traumatic event and persist for a minimum of 3 days and a maximum of 4 weeks. For a diagnosis of PTSD, symptoms must endure for at least one month. While the symptom categories are similar for ASD and PTSD, the number of symptoms required for diagnosis differs. The questionnaires used for this survey included the Primary Care PTSD Screen for DSM-5 (PC-PTSD-5) and the General Anxiety Disorder 7-item (GAD-7), along with sections on sociodemographic information and experiences related to January events.

The PC-PTSD-5 is a 5-item tool developed by the National Center for PTSD in the United States for identification of individuals with probable PTSD at the level of primary healthcare. The tool evaluates the presence and severity of PTSD symptoms over the past month regarding a traumatic exposure event in terms of experience of nightmares, trying hard not to think about it, being constantly on guard, feeling numb or detached from others, and feeling guilty. A cut-point of 4 is considered to be applicable for the overall sample for men, while a lower cut-off value is proposed for women [10].

The GAD-7 is a 7-item screen that is internationally used to identify individuals with probable generalized anxiety disorder. The respondents are asked to answer how often they felt anxious and afraid, experienced restlessness and had difficulty with relaxation, were unable to control worries and were easily annoyed over the last 2 weeks. The severity of symptoms is evaluated by assigning scores of 0, 1, 2 or 3 to the following response categories: “not at all,” “several days,” “more than half the days,” and “nearly every day.” Individuals who obtain scores of 0–4 are considered to be minimally anxious, and scores of 5, 10, and 15 represent mild, moderate, and severe anxiety, respectively [11]. The validated versions of the GAD-7 and PC-PTSD-5 have successfully been utilized in studies involving populations in Kazakhstan [12,13] and Russia [14].

For the purpose of this study, the official Russian versions of the PC-PTSD-5 and GAD-7 were used.

### 2.4 Covariates

Assessment of symptoms associated with post-traumatic stress and anxiety was conducted in relation to several covariates: age, gender and marital status of respondents, having children, employment status, education level, and region of residence. Age groups were classified in accordance with the recommenced standard international age classifications for demographic, social, and related economic data [15] as follows: ≤24, 25–44, 45–64, and ≥65 years old. Gender was categorized as male and female. Marital status was classified as: single, married, divorced, and widowed, while the presence of children was scored as present or absent. Employment status was evaluated as: unemployed, employed, retired, college or university student, and education level was recorded as secondary, secondary vocational, and higher.

### 2.5 Statistical analyses

The Kolmogorov-Smirnov test was used to assess the normality of the data distribution for all continuous variables. Since the data distribution was found to be normal, the data were presented as mean (M) and standard deviation (SD). Descriptive statistics were computed for both continuous and categorical variables. Categorical variables were presented as frequencies and percentages, and the Pearson's χ2 test was employed to determine the statistical significance of differences. The association between demographic and other relevant risk factors and the severity of symptoms according to the PC-PTSD-5 and GAD-7 scales was evaluated using logistic regression models to derive odds ratios (ORs) and 95 % confidence intervals (CIs). A critical value of p < 0.05 was considered significant. All statistical analyses were processed with the help of SPSS (Statistical Package for the Social Sciences) software, version 20.0 for Windows.

## 3 Results

Table 1 presents socio-demographic characteristics of study participants. Most respondents (70.6 %) were females. The mean age of respondents was 30 years. Although most of the study participants (53.2 %) were single, 57.7 % had children. Only 26.1 % of respondents reported directly experiencing the January 2022 events in person, and 11.5 % reported having relatives or friends who were directly affected in person by the unrest. Only 1.7 % experienced property damage during the events.

**Table 1:** Sociodemographic data of study participants (n = 6510).

Characteristics		Number	%
Gender	Female	4595	70.6
	Male	1915	29.4
Age, years	Mean ± Standard Deviation	30 ± 13	
	<25	3516	54.0
	25–44	1880	28.9
	45–64	1018	15.6
	≥65	96	1.5
Marital status	Single	3462	53.2
	Married	2610	40.1
	Divorced	296	4.5
	Widowed	142	2.2
Presence of children	Present	3754	57.7
	Absent	2756	42.3
Education level	Secondary	1808	27.8
	Secondary vocational	650	10.0
	Higher	4052	62.2
Employment status	Unemployed	405	6.2
	Employed	2602	40.0
	College student	107	1.6
	University student	3299	50.7
	Retired	97	1.5
Were you affected by the January 2022 unrest?	No	4808	73.9
	Yes	1702	26.1
Were any of your relatives or friends affected by the January 2022 unrest?	No	5760	88.5
	Yes	750	11.5
Was your property damaged by the January 2022 unrest?	No	6398	98.3
	Yes	112	1.7

Because more than half of the study participants (54.0 %) were younger than 25 years, Table 2 provides details on the severity of symptoms in individuals under the age of 25 years in comparison with people aged 25 years and older. For both cut-points (≥3 and ≥4) on the PC-PTSD-5, the proportion of individuals experiencing post-traumatic stress symptoms was significantly higher in those aged 25 years and older. Similarly, with the GAD-7, for a cut-off of 10, the proportion of individuals experiencing anxiety symptoms was also higher in individuals aged ≥25 years.

**Table 2:** The prevalence of symptoms related to post-traumatic stress and anxiety in people aged <24 years vs. people aged 25 years and older (n = 6510).

Scale and severity		Age <25 years (n = 3516)		Age ≥25 years (n = 2994)		Test of difference	
		n	%	n	%	χ2	p-value
PC-PTSD-5^a^	Score <3	3159	89.8	2387	79.7	131.3	<0.001
	Score ≥3	357	10.2	607	20.3		
	Score <4	3404	96.8	2809	93.8	33.3	<0.001
	Score ≥4	112	3.2	185	6.2		
GAD-7^b^	Score <10	3325	94.6	2475	82.7	235.8	<0.001
	Score ≥10	191	5.4	519	17.3		
	0-4 (none)	2616	74.4	1451	48.5	510.2	<0.001
	5-9 (mild)	665	18.9	966	32.3		
	10-14 (moderate)	183	5.2	358	12.0		
	15-19 (moderately severe)	47	1.3	197	6.6		
	≥20 (severe)	5	0.1	22	0.7		

a PC-PTSD-5 – Primary Care Post-Traumatic Stress Disorder Screen for DSM-5.

b GAD-7 – Generalized Anxiety Disorder 7 item.

The population of respondents residing in Almaty city and Almaty region had significantly higher rates of symptoms associated with both post-traumatic stress and generalized anxiety disorder compared with people residing in other cities and regions of Kazakhstan. For PC-PTSD-DSM-5 cut-off ≥3 as many as 30.0 % of Almaty dwellers had post-traumatic stress symptoms, while for a cut-off ≥4, the proportion was 10.0 %. The nationwide prevalence of post-traumatic stress symptoms among overall respondents constituted 14.8 % for a cut point of ≥3 and 4.6 % for a cut point of ≥4. Likewise, the population of Almaty city and Almaty region had significantly higher prevalence of anxiety symptoms in contrast with the population of other Kazakhstan's regions. For a cut-off point of ≥10 on the GAD-7, 28.2 % of residents in Almaty city and Almaty region experienced anxiety symptoms, as opposed to 10.9 % of individuals residing in other regions of Kazakhstan. (Table 3).

**Table 3:** The prevalence of symptoms related to post-traumatic stress and anxiety in residents of Almaty and Almaty Region vs. population of other regions of Kazakhstan.

Scale and severity		Almaty city and Almaty Region (n = 1001)		Other regions (n=5509)		Total (n= 6510)		Test of difference	
		n	%	n	%	n	%	χ2	p-value
PC-PTSD-5^a^	Score <3	701	70.0	4845	87.9	5546	85.2	215.6	<0.001
	Score ≥3	300	30.0	664	12.1	964	14.8		
	Score <4	900	89.9	5313	96.4	6213	95.4	83.0	<0.001
	Score ≥4	101	10.1	196	3.6	297	4.6		
GAD-7^b^	Score <10	753	75.2	5047	91.6	5800	89.1	234.2	<0.001
	Score ≥10	248	24.8	462	8.4	710	10.9		
	0-4 (none)	375	37.5	3692	67.0	4067	62.5	402.2	<0.001
	5-9 (mild)	344	34.4	1287	23.4	1631	25.1		
	10-14 (moderate)	179	17.9	362	6.6	541	8.3		
	15-19 (moderately severe)	97	9.7	147	2.7	244	3.7		
	≥20 (severe)	6	0.6	21	0.4	27	0.4		

a PC-PTSD-5 – Primary Care Post-Traumatic Stress Disorder Screen for DSM-5.

b GAD-7 – Generalized Anxiety Disorder 7 item.

**Fig. 1:**
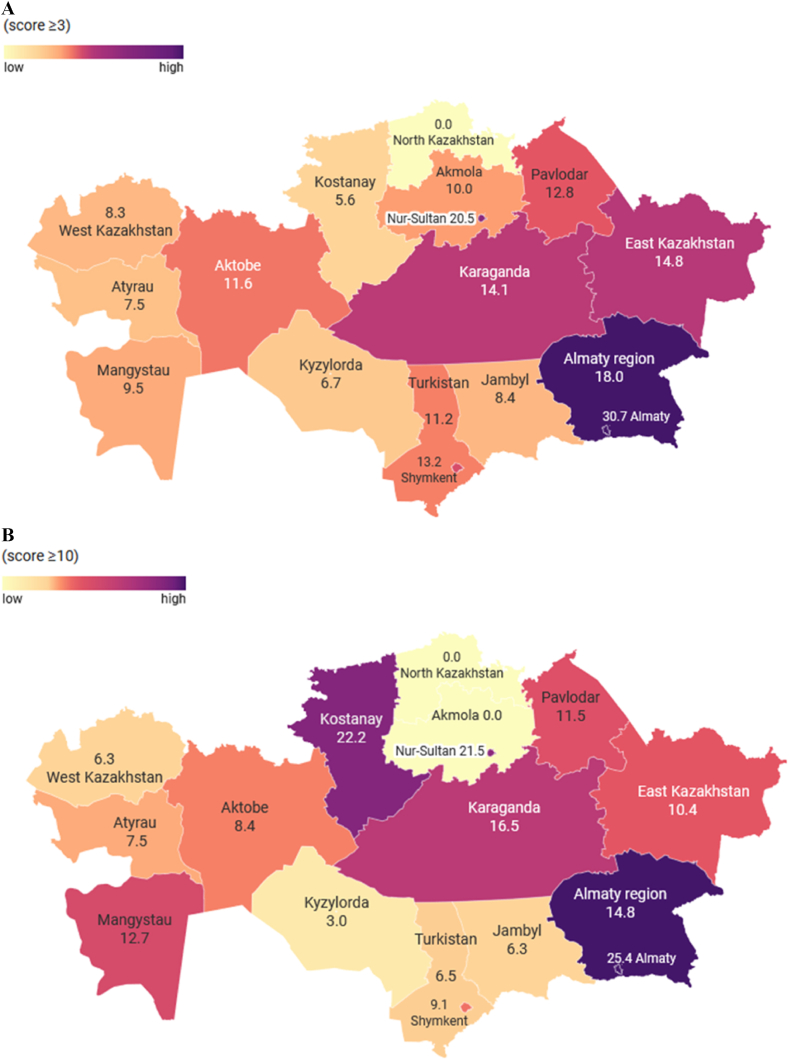
The regional variation in the prevalence of symptoms associated with (A) post-traumatic stress (score ≥3 on the PC-PTSD-5); and (B) anxiety (score ≥10 on the GAD-7).

Fig. 1A and B depict the regional variation in the prevalence of symptoms associated with post-traumatic stress and anxiety, respectively. It was no surprise that for a cut-off ≥3 on the PC-PTSD-5, Almaty city and Almaty region had the highest prevalence of symptoms, followed by Kostanay, East Kazakhstan, and Karaganda regions (16.7 %, 14.8 % and 14.1 %, respectively). Meanwhile, western provinces of the country – Kyzylorda, Atyrau, West Kazakhstan, and Mangystau – were characterized by lower prevalence of symptoms as compared with other regions (6.7 %, 7.5 %, 8.3 % and 9.5 %, correspondingly). Of interest is the fact that for a cut-off ≥10 on the GAD-7, the highest prevalence of anxiety symptoms was seen in Kostanay region (33.3 %), followed by Almaty city (25.4 %), and Karaganda region (16.5 %). Similar to post-traumatic stress symptoms, the population of Kyzylorda region demonstrated the lowest rate of anxiety symptoms (3.0 %).

Table 4 presents the multivariate logistic regression analysis models examining the prevalence of symptoms related to post-traumatic stress and anxiety in relation to the study covariates. In comparison to women, men had significantly lower odds of scoring ≥3 on the PC-PTSD-5 and ≥10 on the GAD-7. Divorced respondents had a higher chance of scoring ≥10 on the GAD-7 (OR = 1.77). The chance of having post-traumatic stress symptoms was twofold higher in individuals who were personally affected by the events, and more than twofold higher in those who had relatives and friends affected by the unrest or those whose property was damaged. The chance to have high scores (≥10) on the GAD-7 was significantly increased only among those respondents whose relatives or friends were directly affected by the January 2022 events.

**Table 4:** Multivariate logistic regression analysis models examining the prevalence of symptoms related to post-traumatic stress (score ≥3 on the PC-PTSD-5) and anxiety (score ≥10 on the GAD-7) by the study covariates (n = 6510).

Covariates		Post-traumatic stress symptoms			Anxiety symptoms		
		OR	95 % CI	p-value	OR	95 % CI	p-value
Gender	Female	reference			reference		
	Male	0.42	0.34–0.51	<0.001	0.67	0.54–0.84	<0.001
Age, years	<25	reference			reference		
	25–44	1.92	1.41–2.61	<0.001	1.59	1.10–2.28	0.01
	45–64	1.72	1.19–2.49	<0.001	2.40	1.59–3.63	<0.001
	≥65	1.28	0.60–2.75	0.52	2.89	1.43–5.84	<0.001
Marital status	Single	reference			reference		
	Married	1.18	0.90–1.55	0.22	1.02	0.75–1.39	0.91
	Divorced	1.17	0.79–1.74	0.42	1.77	1.18–2.68	0.01
	Widowed	0.91	0.53–1.57	0.73	1.13	0.65–1.96	0.67
Presence of children	Present	0.76	0.57–1.01	0.06	0.89	0.64–1.23	0.47
	Absent	reference			reference		
Education level	Secondary	reference			reference		
	Secondary vocational	1.11	0.81–1.53	0.52	0.94	0.63–1.40	0.76
	Higher	1.31	0.98–1.75	0.06	1.33	0.95–1.86	0.10
Employment status	Unemployed	reference			reference		
	Employed	0.85	0.62–1.17	0.32	0.74	0.53–1.02	0.07
	College student	0.99	0.49–2.01	0.97	0.59	0.25–1.40	0.23
	University student	0.81	0.57–1.14	0.22	0.48	0.33–0.71	<0.001
	Retired	0.65	0.31–1.37	0.26	1.50	0.79–2.85	0.22
PC-PTSD-5^a^	Score <3	–	–	–	reference		
	Score ≥3	–	–	–	6.72	5.60–8.06	<0.001
GAD-7^b^	Score <10	reference			–	–	–
	Score ≥10	6.74	5.62–8.10	<0.001	–	–	–
Were you affected by the January 2022 unrest?	No	reference			reference		
	Yes	2.00	1.69–2.37	<0.001	1.18	0.96–1.44	0.11
Were any of your relatives or friends affected by the January 2022 unrest?	No	reference			reference		
	Yes	2.25	1.85–2.76	<0.001	2.12	1.70–2.65	<0.001
Was your property damaged by the January 2022 unrest?	No	reference			reference		
	Yes	2.42	1.52–3.85	<0.001	1.30	0.76–2.20	0.34

a PC-PTSD-5 – Primary Care Post-Traumatic Stress Disorder Screen for DSM-5.

b GAD-7 – Generalized Anxiety Disorder 7 item.

## 4 Discussion

Using an online framework and internationally established structured questionnaires this study aimed to evaluate and compare the prevalence of symptoms associated with post-traumatic stress and anxiety among Kazakhstan population in the aftermath of the January 2022 civil unrests. The study is unique in that it aimed to capture the prevalence of symptoms among the respondents quite proximal to the events. Nationally, for a cut-off ≥3 on the PC-PTSD-5, the prevalence of post-traumatic stress symptoms was 14.8 %, while for a cut-off ≥4, the prevalence was 4.6 %. The population of Almaty city and Almaty region that experienced the most violent disturbances had the highest prevalence of post-traumatic stress symptoms (30.0 % for a cut-off point of ≥3 or 10.1 % for a cut-off ≥4). Thus, the study revealed the substantial prevalence of acute stress reactions in Kazakhstan population that could warrant advising local authorities about implementation of targeted interventions.

Generally, the proximity to the site of violence is an important risk factor for mental distress. This is particularly important for those civil disorders that are associated with fatalities. For example, following Egypt's Arab Spring, the prevalence of PTSD symptoms among students in schools located within 1 km of Tahrir Square – the site of the most violent events – was 70 % and around 60 % of students reported anxiety symptoms [16]. Those individuals who were directly exposed to looting, arson and physical trauma, showed the highest rates of PTSD [17]. Being a victim of a direct attack is also related to PTSD [18], while a personal participation in non-violent mass sociopolitical movement is not associated with a probable anxiety [19]. In the current study, being personally affected by the January 2022 unrest was associated with a twofold increase in the odds of presenting with the post-traumatic stress symptoms, while having relatives or friend affected by the events increased the chance by 2.25 times.

It has been well established that living in areas of civil unrest is associated with exacerbation of psychiatric symptoms [20]. A recent systematic review has emphasized that protests, riots, and revolutions, even when nonviolent can be associated with adverse mental health outcomes, with prevalence of PTSD ranging from 4 %, to 41 % in riot affected areas, the latter figure being consistent with the findings from Almaty city and Almaty region [21]. Since there is usually an increased coverage of social disturbances by local media, a generated spillover effect may induce and deteriorate mental distress. For example, according to Galovski et al., the media exposure and reactions to media were significantly associated with the symptoms of PTSD in individuals who lived or worked within 30 miles of Ferguson, Missouri – a site of the 2014 community protests [22]. In Kazakhstan, internet was disrupted at the peak of the unrest and the public was largely informed by the president's television addresses. To a certain extent, information constraints give rise to gossips and rumours that may further affect mental health in susceptible individuals. Thus, provision of timely and adequate information is one of the pillars of PTSD prevention.

Other risk factors that influence the rates of mental distress after civil riots are unemployment and poor social cohesion [21]. Social cohesion is the ability to maintain strong relations grounded in solidarity and mutual support. Social cohesion is an intrinsic part of the civil society, which is just developing in Kazakhstan [23] and thus, the strength of social interactions could be considered as insufficient. Still, this study did not account for social cohesion and it is not possible to conclude about its protective or harmful role for mental distress following the January 2022 unrest. As for unemployment, there did not appear to be a significant difference between employed and unemployed study subgroups in terms of the prevalence of acute stress reactions. The lack of association between mental health issues and employment status is probably best explained by relatively small numbers in both subgroups. Nevertheless, it has to be noted that the relationship between mental distress and employment is bi-directional as good mental health is crucial for employability and unemployment has negative consequences for psychological health [24].

In the current study, the presence of symptoms associated with post-traumatic stress and anxiety was associated with female gender, older age, being divorced, and having children. Female gender is a well-recognized risk factor for mental health, which is undermined during the period of social disturbances. Moussa et al. reported the rise in prevalence of PTSD symptoms in female school students following the Arab Spring events [25]. Accordingly, female sex was a risk factor for negative change in psychological health in the Hong Kong general public following the Occupy Central movement ^26^. Likewise, in Northern Ireland “troubles”, while males were more likely to have experienced a traumatic event, females were more likely to have PTSD ^27^. Older age was significantly associated with mental distress ^28^. In this study, being divorced was associated with higher rates of symptoms related to post-traumatic stress and anxiety, which was not true for being widowed. This finding is in contrast to other studies that found similar rates of mental distress in these marital status subgroups ^26,29^. Perhaps, the higher rates of symptoms associated with post-traumatic stress and anxiety in divorced individuals could be attributed to a lesser family support traditionally provided to this category of people in Kazakhstan [24]. Also, it was contradictory with the earlier studies to observe the higher rates of acute stress reactions in individuals with children. This is probably because political instability creates uncertainty in the minds of the public considering ability to make ends meet and thus, deteriorates mental health.

This study has several important limitations. First, a major limitation is the unavailability of any baseline data on prevalence of posttraumatic stress and anxiety symptoms in the Kazakhstan population that prevents us from ascertaining the actual changes following the January 2022 unrests. Although this concern could not be overcome at the outset, the study was able to compare prevalence among populations with and without direct exposure to the event. Second, this is a cross-sectional study and thus, we cannot not establish any causality. This leads to inability to evaluate the full mediation effect of different sociodemographic factors on the relationship between riots and perceived symptoms of post-traumatic stress and anxiety. Also, cross-sectional design results in the failure to assess some important confounders, like mental health status before the events. Third, the study comprised an online respondent pool which is not necessarily representative of the general population of Kazakhstan, albeit representing a large sample size. Furthermore, although the respondents were mainly recruited through social media, some were recruited by investigators reaching out to social media sources that may be selective. The respondents therefore do not represent a probability population sample. They included an overrepresentation of younger individuals who are more active users of social media in Kazakhstan compared to older adults. Nevertheless, the approach is useful in studying stigmatized groups experiencing mental distress and can provide an important comparative framework. While efforts were made to create a representative sample, the study acknowledges a potential limitation in fully matching the distribution of age, sex, and living location with that of the entire Kazakh population. Future studies may benefit from calibrating the probabilities to further align the sample characteristics with the broader population. Finally, the screening tools, PC-PTSD-5 and GAD-7, reflect self-report dimensional scales for screening rather than discrete diagnoses of PTSD and GAD and may differ in their discriminating power. On the other hand, the utility of self-administered screening tools in primary care settings is psychometrically sound approach which can be incorporated as economically and readily applicable instruments in primary care practices.

## 5 Conclusion

Social unrest is one of the triggers of emotional distress in the general public. Thus, health authorities of riot-affected areas should give more attention and consider the provision of targeted population-based mental health interventions. Such programs need to focus on identification of individuals with acute stress reactions and include timely delivery of adequate information about common symptoms and associated problems. Management of individuals with emotional distress has to be envisaged via counseling and psychological services. Besides, healthcare professionals should be aware of emotional sequelae after social unrest that may be potentiated by community spillover effects. There is therefore need for further research to address the gaps in knowledge regarding population level mental health in post-conflict communities. Finally, a peaceful resolution has to be found by all sides of the conflict as according to the World Health Organization, “health is one of the fundamental rights of every human being without distinction of race, religion, political belief, economic or social condition” ^30^.

## Funding

This study received no funding. KM was supported, in part, by the NCD-LIFESPAN 10.13039/100000061Fogarty International Center and 10.13039/100000025National Institute of Mental Health grant at 10.13039/100006823Boston Children's Hospital, United States (D43 TW009680).

## Data availability statement

Data will be made available on a request.

## CRediT authorship contribution statement

**Natalya Glushkova:** Conceptualization, Data curation, Formal analysis, Investigation, Methodology, Project administration, Supervision, Writing – original draft, Writing – review & editing. **Alexandr Ivankov:** Conceptualization, Data curation, Formal analysis, Project administration, Software. **Varvara Trenina:** Methodology, Resources, Writing – original draft, Writing – review & editing. **Ainash Oshibayeva:** Data curation, Investigation, Resources. **Zhanna Kalmatayeva:** Resources, Validation, Writing – review & editing. **Zhanar Temirbekova:** Data curation, Investigation, Methodology. **Saltanat Mamyrbekova:** Investigation, Methodology, Resources. **Kairat Davletov:** Conceptualization, Resources, Writing – original draft, Writing – review & editing. **Zaituna Khismetova:** Investigation, Methodology, Resources. **Almagul Kauysheva:** Data curation, Investigation, Methodology, Resources. **Ardak Auyezova:** Data curation, Investigation, Methodology, Resources, Validation. **Marat Zhanaspayev:** Investigation, Validation, Writing – original draft. **Lyudmila Pivina:** Conceptualization, Investigation, Methodology. **Gulbakit Koshmaganbetova:** Investigation, Software, Validation. **Ardak Nurbakyt:** Data curation, Investigation, Methodology, Validation. **Gulzat Sarsenbayeva:** Data curation, Investigation, Methodology, Resources. **Zhanat Sadibekova:** Data curation, Investigation, Methodology, Resources. **Meruert Gazaliyeva:** Data curation, Investigation, Methodology, Resources. **Mukhtar Kulimbet:** Investigation, Methodology, Resources. **Diana Kalmatayeva:** Data curation, Formal analysis, Investigation, Methodology, Resources, Writing – review & editing. **Aliya Zhylkybekova:** Data curation, Investigation, Methodology, Resources, Validation. **Zhansaya Nurgaliyeva:** Data curation, Formal analysis, Investigation, Methodology. **Kassymkhan Sultanbekov:** Data curation, Resources, Validation. **Daniil Semenov:** Conceptualization, Data curation, Formal analysis, Project administration, Software, Writing – original draft, Writing – review & editing. **Marina Izmailovich:** Data curation, Formal analysis, Investigation, Validation. **Oxana Tsigengagel:** Data curation, Formal analysis, Writing – review & editing. **Kerim Munir:** Conceptualization, Supervision, Writing – original draft, Writing – review & editing.

## Declaration of competing interest

The authors declare that they have no known competing financial interests or personal relationships that could have appeared to influence the work reported in this paper.
