# Supplementary material for: Post-conflict acute stress reactions in Kazakhstan in the aftermath of January 2022 unrests: A national survey
Source: Heliyon. 2023 Oct 21;9(11):e21065. doi: 10.1016/j.heliyon.2023.e21065 (PMC10641126; doi:10.1016/j.heliyon.2023.e21065)
Supplement: Multimedia component 1 [file mmc1.docx]

**The Questionnaire: Post-Conflict post-traumatic and generalized anxiety disorders in Kazakhstan in the aftermath of January 2022 unrests: A national survey**

**SOCIODEMOGRAPHIC ITEMS**

1. **Please indicate your gender**
   - Male
   - Female
2. **Please indicate your age (years) _____**
3. **Please, indicate your marital status**
   - Single
   - Married
   - Divorced
   - Widowed
4. **How many children do you have?_____**
5. **Please indicate your employment status**
   - Employed (official permanent job)
   - Unemployed (no official permanent job)
   - Retired
   - University student
   - College student
6. **Please indicate your last completed education**
   - Secondary
   - Secondary vocational
   - Higher
7. **City of residence___________________________**

| **EXPERIENCE OF JANUARY EVENTS** | | | | | | |
| --- | --- | --- | --- | --- | --- | --- |
| **8.** | **Were you affected by the January 2022 unrest?** | | | | Yes | No |
|  | Were any of your relatives or friends affected by the January 2022 unrest? | | | | Yes | No |
|  | Was your property damaged by the January 2022 unrest? | | | | Yes | No |
| **THE PRIMARY CARE PTSD SCREEN FOR DSM-5 (PC-PTSD-5)**  In the past month, have you … | | | | | | |
| **9.** | Had nightmares about the event(s) or thought about the event(s) when you did not want to? | | | | Yes | No |
| **10.** | Tried hard not to think about the event(s) or went out of your way to avoid situations that reminded you of the event(s)? | | | | Yes | No |
| **11.** | Been constantly on guard, watchful, or easily startled? | | | | Yes | No |
| **12.** | Felt numb or detached from people, activities, or your surroundings? | | | | Yes | No |
| **13.** | Felt guilty or unable to stop blaming yourself or others for the events(s) or any problems the event(s) may have caused? | | | | Yes | No |
| **THE GENERAL ANXIETY DISORDER-7 (GAD-7)**  Over the last 2 weeks, how often have you been bothered by the following problems? | | | | | | |
| **14.** | Feeling nervous, anxious or on edge | Not at all | Several days | More than half the days | Nearly every day | |
| **15.** | Not being able to stop or control worrying | Not at all | Several days | More than half the days | Nearly every day | |
| **16.** | Worrying too much about different things | Not at all | Several days | More than half the days | Nearly every day | |
| **17.** | Trouble relaxing | Not at all | Several days | More than half the days | Nearly every day | |
| **18.** | Being so restless that it is hard to sit still | Not at all | Several days | More than half the days | Nearly every day | |
| **19.** | Becoming easily annoyed or irritable | Not at all | Several days | More than half the days | Nearly every day | |
| **20.** | Feeling afraid as if something awful might happen | Not at all | Several days | More than half the days | Nearly every day | |
